# How hospital accreditation requirements bridge enablers for AI readiness: interpretative analysis of intersections in framework standards

**DOI:** 10.3389/fdgth.2026.1769181

**Published:** 2026-03-11

**Authors:** Ericles Andrei Bellei, Raquel Debon, Elísio Costa, Ana Carolina Bertoletti De Marchi

**Affiliations:** 1 Graduate Program in Human Aging, Institute of Health, University of Passo Fundo (UPF), Passo Fundo, Brazil; 2 RISE-HEALTH, Competence Center for Active and Healthy Ageing, Faculty of Pharmacy, University of Porto (U.Porto), Porto, Portugal; 3 Graduate Program in Applied Computing, Institute of Technology, University of Passo Fundo (UPF), Passo Fundo, Brazil

**Keywords:** accreditation, artificial intelligence, digital transformation, governance, health services administration, hospitals, implementation science, organizational maturity

## 1 Introduction

Healthcare services have been shaped by a dynamic interplay between established quality paradigms and disruptive technological innovation (1, 2). On the one hand, artificial intelligence (AI) is increasingly viewed as a key driver of healthcare transformation (3, 4), with the potential to improve clinical decision-making, operational efficiency, and patient outcomes (5, 6). In parallel, the enduring pursuit of quality and safety has been institutionalized through healthcare accreditation (7). Accreditation frameworks act as a cornerstone of organizational practice by providing structured approaches to patient safety, process standardization, and continuous quality improvement (8). In Brazil, the National Accreditation Organization (*Organização Nacional de Acreditação*—ONA) offers a rigorous methodology recognized by the International Society for Quality in Health Care for evaluating healthcare service delivery (9), and it is widely adopted by healthcare organizations (10).

Although AI implementation and accreditation are often pursued as distinct strategic initiatives, a more integrated perspective is warranted. We argue that the requirements embedded in accreditation frameworks can help build foundational organizational readiness for effective AI adoption. In other words, compliance with accreditation standards may support the governance structures and process maturity needed to leverage AI in healthcare services. Accordingly, we present an interpretive analysis mapping requirements from the ONA manual against AI implementation enablers and barriers synthesized in the 2024 systematic review by Rahimi et al. (11). By examining intersections across governance, data infrastructure, process maturity, and human factors, we show how accreditation can support a hospital’s broader digital transformation journey.

## 2 Intersections between accreditation requirements and AI readiness factors

We adopted an interpretive approach to examine the alignment between hospital accreditation requirements and organizational readiness for AI implementation. Our analysis drew on two primary sources: (i) the Brazilian National Accreditation Organization (ONA) Accreditation Manual (2022–2026 edition) (9) and (ii) the 2024 systematic review by Rahimi et al. (11), which synthesizes enablers and barriers to AI implementation in hospital settings.

The analysis followed three steps. First, AI enablers and barriers were extracted and grouped according to their original classification into People, Process, Information, and Technology dimensions. Second, the ONA standards were reviewed in full, with particular attention to sections addressing governance, leadership, quality and safety management, information systems, technology management, and workforce development. Third, we conducted an interpretive mapping to identify conceptual correspondences and shared organizational mechanisms, routines, and capabilities rather than one-to-one equivalence.

**Figure 1:**
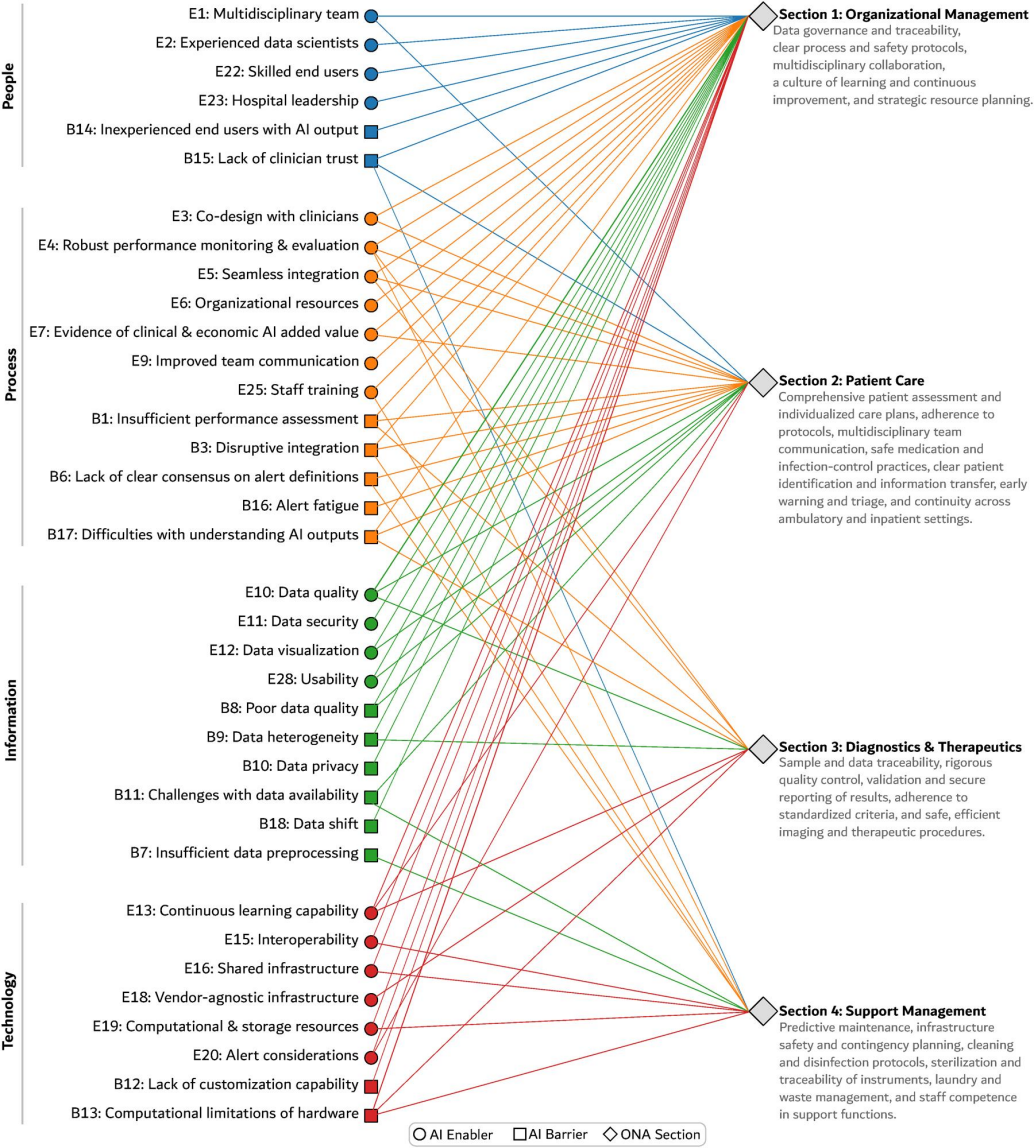
Clustered bipartite network illustrating the alignment between ONA accreditation requirements and enablers and barriers for AI implementation.

This analysis was intentionally exploratory and theory-informed, aiming to generate conceptual insights while acknowledging that accreditation frameworks were not designed with AI implementation as an explicit objective. A simplified schematic representation of the identified connections is shown in Figure 1. Although accreditation is not a direct roadmap for AI, its principles can help build essential organizational capabilities, as we discuss below.

### 2.1 Foundational governance and strategic leadership: creating a mandate for change

Strategic governance and decisive leadership form the bedrock of major organizational transformation, and the integration of AI is no exception. This domain is critical for articulating a vision, allocating resources, and creating an institutional mandate for change. In this regard, ONA’s leadership standards—particularly those in *Subsection 1.1, Organizational Leadership*—align with the *People* and *Process* enablers for AI identified by Rahimi et al. (11).

The ONA manual requires hospital leadership to establish formal strategic planning (Subsection 1.1, Level 1, Req. 3), to create documented institutional policies for critical areas such as information management, quality, and patient safety (Reqs. 8 and 10), and to clearly define organizational structures and responsibilities (Req. 19). This structured, top-down approach is conducive to launching and sustaining complex, resource-intensive initiatives such as AI implementation. In non-accredited institutions, by contrast, AI initiatives may emerge from departmental silos and lack the executive mandate—and resource protection—needed to survive budget cycles and internal politics.

Moreover, the discipline of creating and maintaining documented policies (Reqs. 8, 10, 11, and 12) establishes routines for documentation, version control, and audit trails that are essential for AI model governance, validation, and (where applicable) regulatory submissions to bodies such as ANVISA or the FDA. These practices support the formal allocation of *organizational resources* (Enabler 6) and foster cross-departmental collaboration, which is essential for forming a *multidisciplinary team* (Enabler 1) capable of bridging clinical, technical, and administrative domains. While governance sets strategic direction, AI readiness ultimately also depends on the quality and security of the underlying data and technological infrastructure, which ONA addresses as well.

### 2.2 Data and technology infrastructure: building the digital foundation

AI is fundamentally data-dependent. Its success hinges on a robust technological infrastructure capable of collecting, storing, and processing high-quality data. ONA’s requirements for technology and information management—primarily outlined in *Subsection 1.7, Technology Management and Information Security*—align with the *Information* and *Technology* dimensions of AI readiness identified by Rahimi et al. (11).

To address data-related barriers, ONA requires hospitals to establish methods for data collection and analysis to support decision-making (Subsection 1.7, Level 1, Req. 7) and to implement information security rules (Req. 8). These requirements directly relate to key implementation barriers, including *poor data quality* (Barrier 8) and *data heterogeneity* (Barrier 9). By promoting standardized data governance, accreditation helps strengthen the data assets that AI models depend on.

In addition, the framework’s insistence on defining criteria for acquiring and incorporating new technologies—developed by a *multidisciplinary team* (Req. 17)—supports the development of a *shared infrastructure* (Enabler 16) and reinforces the need for a *multidisciplinary team* (Enabler 1). Coupled with the requirement for systematic maintenance of information systems (Req. 14), these standards reduce ad hoc adoption of incompatible systems and encourage scalable technology growth, thereby supporting *computational and storage resources* (Enabler 19).

Finally, ONA’s requirement to establish a data governance program based on a security platform that specifies data privacy and transaction flows (Req. 11) provides a foundation for interoperability (Enabler 15) and data security (Enabler 11). These capabilities are relevant for integrating AI tools with medical record systems and for meeting data protection requirements, thereby mitigating the data privacy barrier (Barrier 10).

### 2.3 Process maturity and continuous improvement: cultivating a learning culture

Once a robust technological foundation is established, attention shifts to the maturity of organizational processes. The principles of continuous improvement embedded in accreditation are critical to the long-term success of AI (12). Non-accredited organizations may pursue one-off AI implementations, leaving models vulnerable to performance decay. In contrast, ONA-accredited institutions are oriented toward a continuous *monitor–evaluate–improve* cycle. This process-centric approach—embedded in *Subsection 1.2, Quality and Safety Management* and reflected in the requirements for Level 3 (excellence in management)—cultivates key competencies for governing AI systems post-deployment, including:

Systematic monitoring as a precursor to AI evaluation: ONA’s Level 2 requirement to establish a systematic method for data collection, goal definition, and analysis of results to support decision-making (Subsection 1.2, Level 2, Req. 3) provides mechanisms that can be extended to *robust performance monitoring and evaluation* (Enabler 4) of AI models. Organizations accustomed to tracking clinical and operational KPIs are better positioned to monitor AI performance, accuracy, and downstream impact.

Culture of innovation and change management: Achieving ONA’s Level 3 requires demonstrating an *innovative, proactive, and integrated management model* (Level 3, Req. 1). This is operationalized through requirements to *promote improvement cycles based on the results of protocols and committees* (Level 3, Req. 4), fostering an iterative test–learn–adapt cycle that supports AI optimization and enables the development of *new models of care* [Horizon 3 in Rahimi et al. (11)].

Proactive risk management for AI governance: ONA’s requirement to implement a plan for identifying, analyzing, and treating risks (Subsection 1.2, Level 1, Req. 4) parallels processes needed for responsible AI governance. Because AI models can degrade over time (e.g., *data drift* or *data shift*; Barrier 18), organizations with mature risk management structures already have committees and reporting mechanisms (e.g., quality and safety committees) that can be adapted to oversee AI performance. This creates a governance home for post-deployment surveillance and supports managing *data shift* (Enabler 8).

### 2.4 Human factors and workforce readiness: building capacity and trust

Ultimately, the success of processes and technologies rests on the people who use them (13), making workforce readiness a crucial component of AI implementation. Technology alone is insufficient for transformation; effective AI integration is a socio-technical challenge that depends on the clinical workforce’s capacity and willingness to adopt new tools (5, 14).

ONA’s personnel management requirements, detailed in *Subsection 1.5, People Management*, support the skills and culture needed for effective AI adoption. This contrasts with non-accredited settings, where new tools may be delegated to unprepared staff, fostering distrust and poor uptake. In particular, these requirements align with *skilled end-users* (Enabler 22) and *staff training* (Enabler 25) identified by Rahimi et al. (11). Standards requiring organizations to identify training needs (Subsection 1.5, Level 1, Req. 8) and to implement continuous education programs (Req. 10) help mitigate two major barriers: *inexperienced end users* (Barrier 14) and *lack of clinician trust* (Barrier 15).

More broadly, organizations that comply with these standards are already committed to a continuous cycle of assessing competency gaps and delivering targeted education (15), which can be adapted to include digital literacy and AI-specific competencies. Finally, the accreditation-driven emphasis on evidence-based practice (a recurring tenet across the ONA manual) can strengthen clinician trust by encouraging critical engagement with the evidence underpinning AI recommendations rather than reflexive acceptance or rejection (16). Together, these intersections across governance, technology, process, and people suggest that, although not designed for AI, accreditation can help build a holistic foundation for organizational readiness.

## 3 Discussion: accreditation (also) as a pathway for AI implementation

In this analysis, we highlighted key intersections between healthcare accreditation requirements and prerequisites for successful AI implementation. We argue that the rigorous, process-oriented framework of ONA accreditation can function as a pathway to AI readiness in hospitals. Although our analysis focuses on the Brazilian accreditation framework, many of the mechanisms identified—including formal governance structures, standardized data management practices, continuous quality improvement cycles, and systematic workforce training—are also common across international accreditation models such as Joint Commission International, the National Accreditation Board for Hospitals (NABH), and Accreditation Canada. Therefore, the conceptual insights presented here may be transferable to other healthcare systems operating under comparable accreditation regimes.

Nevertheless, caution is warranted when extrapolating these findings to contexts with less formalized governance structures or more fragmented regulatory environments. The strength of the alignment between accreditation and AI readiness is likely to vary by national context. Moreover, accreditation compliance does not guarantee AI success and should not be treated as a substitute for a dedicated, context-specific AI strategy. While accreditation can build foundational readiness, organizations must still develop AI-specific governance policies, validation procedures, and ethical frameworks.

This interpretive analysis opens avenues for future research. Empirical studies comparing AI adoption outcomes between accredited and non-accredited hospitals could test the hypothesis that accreditation-driven organizational maturity enhances AI readiness. Longitudinal case studies may clarify how accreditation processes interact with the AI lifecycle, from pilot implementation to large-scale deployment and post-deployment monitoring. Finally, accreditation bodies could consider developing supplementary guidance or optional standards addressing digital maturity and AI governance, thereby strengthening accreditation as a strategic instrument for the safe, effective, and ethical integration of AI into healthcare delivery.
